# In Vitro Estimation of the Effect of Grinding on Rumen Fermentation of Fibrous Feeds

**DOI:** 10.3390/ani10040732

**Published:** 2020-04-23

**Authors:** Ignacio Rubén Ortolani, Zahia Amanzougarene, Manuel Fondevila

**Affiliations:** Departamento de Producción Animal y Ciencia de los Alimentos, Instituto Agroalimentario de Aragón (IA2), Universidad de Zaragoza-CITA, M. Servet 177, 50013 Zaragoza, Spain; nacho_ortolani@hotmail.com (I.R.O.); zahiaagro@yahoo.fr (Z.A.)

**Keywords:** in vitro fermentation pattern, fibrous sources, non-processed, ground, gas production

## Abstract

**Simple Summary:**

Intensive feeding systems for beef production are based on high proportions of concentrate at the expense of forages, which can lead to digestive disorders. However, the particle size of the different fibrous feeds can also affect the rumen fermentation pattern, and thus animal performance. Fermentation of six fiber sources (soybean hulls, sugarbeet pulp, palm kernel cake, oat hulls, dehydrated alfalfa meal, and barley straw) in two presentation forms (non-processed and ground) was studied in a closed batch in vitro system. Higher gas production was recorded when substrates were presented in ground form, except for barley straw; however, substrates ranked in the same order irrespective of their presentation form. The particle size did not markedly affect volatile fatty acids proportions. Methane production as an index of fermentation efficiency did not show major differences between feed presentation forms, or non-forage substrates as compared with straw and is related more with the magnitude of fermentation than with qualitative changes in fermentation. Considering other feed components, the comparison of substrates on rumen microbial fermentation depends not only on their fiber proportion but can also be mediated by their levels of protein and fat.

**Abstract:**

The fermentation patterns of six fiber sources, soybean hulls (SH), sugarbeet pulp (BP), palm kernel cake (PK), oat hulls (OH), dehydrated alfalfa meal (DA), and barley straw (BS) were evaluated for this study on the effect of their presentation form (non-processed, NP and ground, GR). Substrates were tested in a conventional in vitro batch system, using rumen fluid obtained from ewes fed 0.5 alfalfa hay and 0.5 barley straw. All substrates rendered a higher gas production in GR form (*p* < 0.05) except for BS but ranked similarly irrespective of the presentation form. Among the substrates, when incubated NP, the highest volume of gas was recorded with BP from 8 h onwards (*p* < 0.05), whereas OH and BS resulted in the lowest gas volume (*p* < 0.05). During the first half of the incubation period, methane production was higher in GR than NP (*p* < 0.05). Among substrates, despite NP or GR, methane production with BP was the highest (*p* < 0.05). Similarly, the presentation form did not qualitatively affect fermentation, as no differences were observed in volatile fatty acids proportions. The effect of particle size of fibrous substrates does not have a major impact on the rate and extent of the rumen microbial fermentation.

## 1. Introduction

Beef cattle feeding in the Mediterranean countries is generally based on the use of high proportions of concentrate feeds, plus cereal straw given in long form. Concentrate and straw are offered separately, and both given ad libitum, reaching proportions of around 90:10 concentrate to straw ratio [1,2,3], in order to reach high animal performance. Under such circumstances, the role of straw as a fibrous source is to maintain a functional rumen environment, although there is a risk of subclinical acidosis and other digestive disorders [4]. The effect of long fiber on the enhancement of mastication, rumination, and salivation [5,6] complements that of dietary proportion of fiber, promoting a healthier rumen environment.

Straw costs are high as its management requires high labor, space availability, and inefficient transport, so other fibrous alternatives have been recently considered. The inclusion of agro-industrial fibrous by-products, with similar proportions of either insoluble (cellulose, hemicelluloses) or soluble (mostly pectin) polysaccharides as forages [7], offered together with the concentrate as a total mixed ration has been proposed as a way to reduce such inconveniences. Non-forage fiber sources are heterogeneous and widely variable in cell wall proportion and composition, as well as in physical characteristics [6,8]. Therefore, their smaller particle size can limit their role as effective fiber in the rumen. Some fibrous by-products can have a particle size over 1.18 mm, which has been considered to be a limiting size for enhancing mastication effect [9], although Yang and Beauchemin [10] found that this effect was promoted by fibrous particles over 8 mm. Recent studies have approached the potential of small particle size fiber sources as forage substitutes [11], and Iraia et al. [12] found that rumen pH and time of mastication and rumination were not correlated with particle size of dietary fibrous feeds.

Despite the effects on animal feeding behavior, particle size has been positively correlated with neutral detergent fiber (NDF) digestibility, associated with higher chewing time, rumen pH, and acetate to propionate ratio [13], although the contrary [14] or no effect [15] have also been reported. In contrast, a smaller particle size should increase microbial access, and thus the rate, although not the extent, of fermentation [16]. Alternatively, a higher fermentation efficiency in terms of lower methane production can be expected when forages are substituted with increasing levels of small particle size by-products [17], partly because of a reduction in acetate proportion and an increased rate of passage of concentrate particles [18].

The in vivo study of responses in rumen fermentation is labor and cost expensive, and it is difficult to differentiate the rate and extent of fiber fermentation from those effects related to animal feeding behavior, such as intake, rate of passage, or salivation. In contrast, in vitro studies can be useful to mimic the fermentation pattern of substrates independently and under controlled conditions, allowing the estimation of digestion kinetics of a single feed [19]. However, as a standardization compromise, feeds are generally incubated ground to 1 mm size [16,20], and thus the in vitro study of particle size effect is seldom considered.

Therefore, this work aimed to study the effect of grinding, on in vitro microbial fermentation, to reduce the particle size of several fibrous substrates differing in their cell wall proportion and composition by comparing them to their natural (non-processed) presentation form in relation to ground to 1 mm particle size, considering cereal straw in long form (20 mm) as a standard feed.

## 2. Materials and Methods

### 2.1. Substrates and Inocula

The following six fiber sources were chosen as substrates for comparison: soybean hulls (SH), sugarbeet pulp (BP), palm kernel cake (PK), oat hulls (OH), dehydrated alfalfa meal (DA), and barley straw (BS). Chemical composition and particle size distribution are given in Table 1. For incubation purposes, BS was manually cut into particles of around 20 mm length, simulating forage intake in ruminants. A subsample of each substrate was ground in a hammer mill (Retsch Gmbh/SK1/417449, Haan, Germany) through a sieve of 1 mm.

Rumen fluid was obtained from four adult ewes (54.5 ± 6.8 kg live weight) housed in the facilities of the Servicio de Apoyo a la Experimentación Animal of the Universidad de Zaragoza. From three weeks before the experiment, donor animals were daily given 1000 g of a 1:1 forage (0.5 alfalfa hay and 0.5 barley straw) to concentrate (0.6 barley grain, 0.20 maize grain, and 0.20 soybean meal) diet, in a single offer at 09:00. On each incubation run, rumen contents (approximately 300 mL) of each animal were sampled before feeding and filtered through a cheesecloth. Then, contents from the four animals were mixed, dispensed in thermos flasks, and immediately transferred to the lab for incubation.

Animal care and procedures for extraction of rumen inoculum were approved by the Ethics Committee for Animal Experimentation (PI12/06). Care and management of animals agreed with the Spanish Policy for Animal Protection RD 53/2013, which complies with EU Directive 2010/63 on the protection of animals used for experimental and other scientific purposes.

### 2.2. Experimental Procedures

The six substrates were incubated in two presentation sizes: non-processed (NP) (as they are commercialized, except for BS that was cut to 20 mm) and ground to 1 mm (GR). Four incubation runs (48 h) were carried out, following the Theodorou et al. [21] procedures, but without microminerals and resazurin [20], with 4 bottles per treatment, plus another 4 bottles without substrate as blanks of inoculum (total of 52 bottles per run). The concentration of bicarbonate buffer in the incubation solution was adjusted to obtain an incubation pH of 6.5, as in Amanzougarene and Fondevila [22]. Incubations were carried out for 48 h in a water bath at 39 ℃. Two bottles per treatment were used to determine gas production, and the pressure was recorded at 2, 4, 6, 8, 10, 12, 16, 24, 36, and 48 h. After that time, the bottles were opened, their pH measured (CRISON micropH 2001, Barcelona, Spain) to validate incubation conditions, and the liquid medium sampled (2 mL on 0.5 mL solution of 0.5 M phosphoric acid with 1 mg 4-methyl-valeric acid as internal standard) and stored at −20 ℃ until analysis of volatile fatty acid (VFA) concentration. The remaining content was filtered through nylon bags (45 µm pore size) that were dried at 60 ℃ for 48 h to determine organic matter disappearance (OMd).

Methane concentration in the gas produced was determined at 0 to 12 h, 12 to 24 h, 24 to 36 h, and 36 to 48 h intervals, by a single sample (5 mL) of the gas produced in the whole interval. Gas samples for the 0 to 12 h and for 12 to 24 h intervals were collected from the third and fourth incubated bottles per treatment, whereas one of the two bottles used for gas production was used for gas sampling of the 24 to 36 h and 36 to 48 h intervals. Liquid medium from the bottle incubated for 12 h was also sampled for the analysis of VFA concentration.

### 2.3. Chemical and Physical Analyses

Dry matter (DM) and organic matter (OM) contents in ground substrates and incubation residues were analyzed following the AOAC [23] procedures (methods ref. 934.01 and 942.05). Substrates were also analyzed for crude protein (CP) and ether extract (EE) (ref. 976.05 and 2003.05) [23], and their concentration of aNDFom was analyzed as described by Mertens [24] in an Ankom 200 Fibre Analyzer (Ankom Technology, New York, NY, USA), using α-amylase and sodium sulphite, and results were expressed exclusive of residual ashes. The ADF (ref. 973.18) and ADL were determined as described by AOAC [23] and Robertson and Van Soest [25], respectively. The estimation of particle size in non-processed substrates was carried out by dry sieving. About 200 g DM of each sample were placed in a sieve shaker fitted with 2.00, 1.20, 0.60, 0.30, and 0.15 mm pore size sieves (Filtra Vibración, Badalona, Spain) and manually shaken in horizontal movements for 40 min. After, sieves were weighed to determine the residual DM on each. For those substrates having particles retained by the 2 mm sieve (OH and SH), the average particle size of such particles was measured under a dissecting microscope. The average particle size was calculated as the weighted mean for each substrate.

Pressure produced on each bottle was measured with a HD8804 manometer provided with a TP804 pressure gauge (Delta OHM, Caselle di Selvazzano, Italy). Readings corrected for the atmospheric pressure were converted to volume (mL) using a pre-established linear regression (n = 103, *R^2^* = 0.996) recorded in the same type of bottles and expressed per unit of incubated OM. Methane concentration was measured in an Agilent 6890 apparatus (Agilent Technologies, Madrid, Spain) equipped with a capillary column (HP-FFAP polyethylene glycol TPA, 30 m × 530 µm id), calibrated with a 10% CH_4_ standard, with a flux of 2 mL/min at 250 ℃. The frozen samples of incubation medium were thawed and centrifuged at 13,000× *g* for 15 min at 4 ℃ for their analysis of VFA, which were determined by gas chromatography on the same apparatus than for methane analysis.

### 2.4. Calculations and Statistical Analyses

Results were analyzed statistically by ANOVA using the Statistix 10 package [26], considering the incubation series as a block. The effect of the nature of presentation (n = 2), the substrate (n = 6), and their interactions were studied as factors for each time of sampling. For total gas production and OMd, the experimental unit was the average of the two bottles per treatment incubated in the same run, whereas for methane production and VFA pattern at 12 h, values from a single bottle per run were considered. Treatment differences among means with *p* < 0.05 and 0.05 < *p* < 0.10 were accepted as representing statistically significant differences and a trend to differences, respectively. When significant, differences were contrasted by the Tukey *t*-test.

## 3. Results

Particle size distribution in NP substrates (Table 1) shows that a high proportion of OH and BP, and to a lower extent SH, were over 1.20 mm (0.79, 0.77, and 0.60, respectively). In contrast, 0.63 of DA particles were below 0.60 mm, whereas PK had major proportions of particles lower than 0.60 mm and higher than 1.2 mm (0.36 and 0.46). BS was not considered in this estimation as it was cut to a fixed size of 20 mm. Mean inoculum pH in the four incubation runs was 6.67 ± 0.23, and averaged 6.59 (from 6.34 to 6.70), 6.53 (6.38 to 6.65) and 6.50 (6.38 to 6.59) at 12, 24, and 48 h.

**Figure 1:**
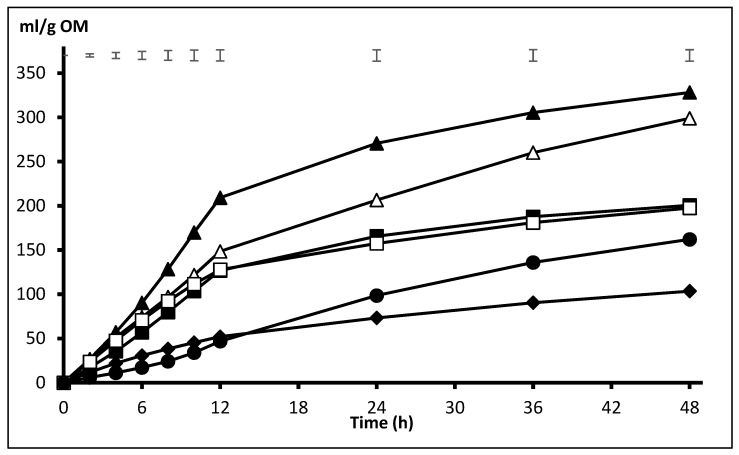
Pattern of gas production from the fibrous substrates (SH △, BP▲, PK ■, OH ♦, DA ☐, and BS ●) incubated non-processed (NP). Upper bars show standard error of means (n = 4).

**Figure 2:**
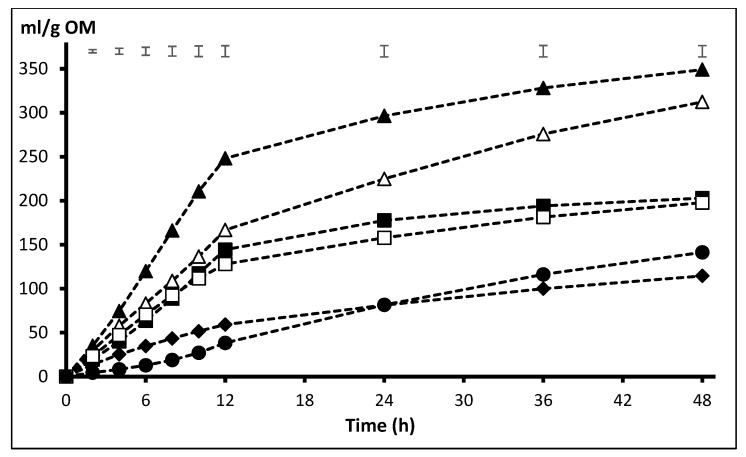
Pattern of gas production from the fibrous substrates (SH △, BP▲, PK ■, OH ♦, DA ☐, and BS ●) incubated ground (GR). Upper bars show standard error of means (n = 4).

The effect of the interaction presentation x substrate on gas production was significant from 6 to 48 h (*p* < 0.05) and tended to be significant at 4 h (*p* = 0.080). This highlights the higher gas production from BP incubated in GR form as compared with NP that was observed from 4 to 12 h (229 vs. 209 mL/g OM at 12 h incubation) and the fact that all substrates produced more gas in ground form (significant effect of presentation form from 2 to 24 h, *p* < 0.05) except BS, that recorded numerically higher values when NP as compared with GR (47 vs. 38, 99 vs. 82, and 162 vs. 141 mL/g OM at 12, 24, and 48 h incubation, *p* > 0.05). For a clearer interpretation of results, these are presented separately for NP and GR in Figure 1 and Figure 2. Among NP substrates (Figure 1), the highest volume of gas was recorded with BP from 8 h onwards, whereas SH did not show any differences as compared with DA and PK until 24 h, but it was higher thereafter. Throughout the incubation, OH and BS had the lowest volume of gas production, but, at 36 and 48 h, gas from BS was higher than OH (*p* < 0.05). Substrates ranked similarly when they were incubated ground (Figure 2), with BP producing the highest gas volume from 6 to 48 h incubation and SH fermenting at a higher extent than DA and PK from, respectively, 12 and 24 h onwards (*p* < 0.05). Again, OH and BS recorded the lowest gas volumes along the whole incubation period.

**Figure 3:**
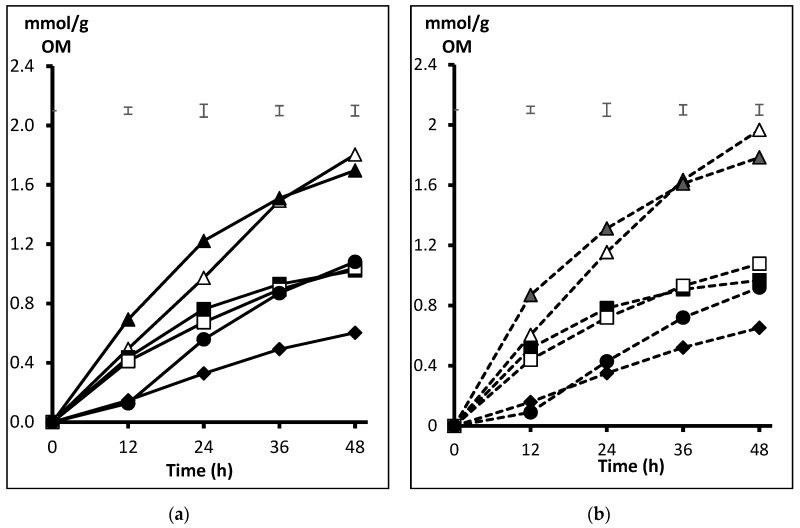
Average pattern of methane production (mmol/g OM) from fibrous substrates (SH △, BP▲, PK ■, OH ♦, DA ☐, and BS ●) incubated non-processed (NP, (**a**)) or ground (GR, (**b**)). Upper bars show standard error of means (n = 4)

When the accumulated methane production was considered (expressed as mmol/g OM of incubated substrate), results in the first half of the incubation period were higher in GR vs. NP (0.45 vs. 0.38 from 0 to 12 h, *p* < 0.001, and 0.79 vs. 0.75 from 12 to 24 h, *p* < 0.022), but differences disappeared thereafter. Since a significant effect of the interaction presentation x substrate was detected at any time interval (*p* < 0.01), substrate comparison is shown separately for NP (Figure 3a) and GR (Figure 3b) presentation forms. Among NP substrates, methane production with BP was the highest up to 24 h incubation, but differences between this and SH disappeared thereafter. Methane became higher with SH than PK, DA, and BS from 24 h onwards, and OH recorded the lowest methane production throughout the incubation period, although it did not differ with BS at 12 h. When incubated GR (Figure 3b), methane from BP was also the highest up to 24 h, but that from SH proportionally increased thereafter and became higher than BP at 48 h. At all incubation times, methane production was also higher with SH than PK, DA, BS, and OH. Again, OH recorded the lowest methane production, except that it did not differ with BS at 12 and 24 h. Methane proportion in total gas produced is shown in Table 2. In the first 12 h, no effect of the presentation form was detected, but OH and BS recorded the overall substrate lowest proportion (*p* < 0.05). From 12 to 24 h no differences were recorded among NP substrates, whereas in GR form methane proportion was higher with SH and DA than PK and BS, and BP was also higher than BS (effect of the interaction presentation x substrate, *p* < 0.013). From 24 to 36 h, PK recorded the lowest methane proportion, and with BS it was lower than SH (*p* < 0.05). Again, substrate response differed between presentation forms in the last fermentation interval (36 to 48 h, *p* = 0.025), and PK rendered less methane than OH and DA when NP but it resulted lower than the other substrates, except for BS, when GR (*p* < 0.05).

The proportion of OMd after 48 h incubation was higher when presented ground (0.628 vs. 0.605 for GR and NP, *p* = 0.029). Among substrates (Table 2), overall OMd means of BP and SH were the highest, whereas OH was the lowest, even below BS (*p* < 0.05). The effect of the interaction presentation form x substrate on OMd was not significant (*p* > 0.10).

Table 3 and Table 4 show no differences between the presentation forms in total VFA concentration nor in molar VFA proportions, at either 12 or 24 h incubation. At 12 h, fermentation of BP produced a higher total VFA concentration than the other substrates (*p* < 0.05) except for DA. Additionally, BP showed the highest proportion of propionate and lowest proportion of branched-chain volatile fatty acids (BCVFA, sum of isobutyrate and isovalerate), whereas PK showed the highest proportion of butyrate at the expense of propionate which was lower than BP and SH, and acetate which was lower than DA. At 24 h incubation, the highest total VFA concentration was observed for BP and SH. Again, PK showed the highest butyrate proportion, whereas those of acetate and propionate were lower than BS, SH, and BP, and lower than BP and DA, respectively. In addition, propionate proportion was higher with BP than BS and OH, and that of butyrate was lower with BS, SH, and BP than with that of OH. The effect of the interaction presentation form × substrate was not significant on any parameter at either 12 or 24 h (*p* > 0.05).

## 4. Discussion

### 4.1. Effect of Particle Size

The proportion and composition of the fibrous fraction of feeds determines its nutritive value, but its physical presentation form that can determine its rate of intake and modulate the digesta rate of passage [13,19] must also be considered. However, for forages, particle size has been generally measured into large categories (more than 19 mm, from 8 to 19 mm, between 1.18 and 8 mm, and less than 1.18 mm) which do not allow for characterizing most non-forage fibrous feeds as such intervals do not fit with their smaller sizes. Thus, Iraia et al. [12] described barley straw, soybean hulls, beet pulp, and whole cotton seeds as having 0.64 to 0.94 of particles smaller than 8 mm, whereas their average particle size was 1.5 to 3.0 mm. In our study, the average particle size ranked substrates in three categories, i.e., over 5 mm (OH), from 1.7 to 1.9 mm (BP and SH), and below 1.2 mm (PK, DA), whereas BS was homogenized by manually cutting to approximately 20 mm.

An in vitro approach does not allow for the study of the effect of presentation form on feeding behavior of ruminants assumed as the proportion of fiber that stimulates chewing and salivation, rumination, and ruminal motility that is referred to as effective fiber (peNDF [27]). Thus, this work is restricted to the evaluation of a potential effect of particle size on fermentation, determined by the physical availability of feed particles to microbial attack. In any case, if peNDF of substrates in their non-processed form is estimated from the amount of NDF and the proportion of particles over 1.2 mm size [9], it should be 0.65, 0.35, 0.35, 0.28, and 0.05 for OH, SH, BP, PK, and DA, respectively. By comparison, peNDF for BS estimated from its NDF content and considering a physical effectiveness factor (pef) of 0.80 [9] should give a value of 0.61, close to that of OH and higher to the other non-forage substrates. It is worth considering that a level of 0.07 to 0.10 of peNDF in feed DM has been recommended for intensively reared beef cattle (8%–10% forage, in DM basis) to maintain rumen pH above 5.7 [28], although it is often below in commercial practice [27].

According to Zebeli et al. [29], large feed particle size could increase resistance to microbial attachment and degradation. In our study, grinding of substrates promoted a higher rate of fermentation estimated from gas production, resulting in a 0.12 increase at 6 h fermentation, which was reduced afterwards to 0.09 and 0.05 after 12 and 24 h incubation, and to only 0.02 after 48 h. Similarly, a higher OMd (*p* < 0.05) was observed with GR substrates, although increased losses of substrate during filtration because of grinding to 1 mm cannot be discarded. The positive effect of grinding on fermentation was not manifested as a clear response on total VFA, although the differences were of a similar magnitude (0.12 times higher for GR than NP at 24 h incubation), partly because of the high magnitude of the error term (coefficient of variation 0.39). The increase in gas volume that was numerically appreciable for SH, PK, and OH (0.11 to 0,12 higher in GR than NP form at 12 h), only resulted significant for BP (*p* < 0.05). Considering the high fermentability of BP [30], reducing particle size from 1.77 to 1.00 mm should increase nutrient accessibility. No effect of grinding was detected on DA, which was expected considering that the average particle size of this substrate was below the size of grinding. However, in the case of BS, a numerically (*p* > 0.10) higher fermentation was observed when incubated NP, with gas volumes 0.07 and 0.21 higher than GR at 12 and 24 h, respectively, which contrasts with the results of coarse and fine grinding roughages observed by Menke and Steingass [16]. Grinding straw could release monophenolic compounds such as ferulic and *p*-coumaric acids, which can depress fibrolytic microbial activity [31,32], but this effect was of minor magnitude. In any case, substrates ranked the same despite their presentation form, and therefore it seems evident that grinding to a standard size of 1 mm is a good alternative for the in vitro study and comparison of the fermentation pattern of feeds [16,20].

Alternatively, the fermentation of non-processed substrates was slightly more efficient than when incubated ground, as the methane proportion in the gas produced tended (*p* = 0.06) to be higher in GR from 36 to 48 h. This should be against the lower methane production expected when feeds are given finely ground as compared with chopped [33,34]. However, the heterogeneous particle size of the non-processed substrates in relation to their ground form, together with the potential responses in their rate of passage [35] that cannot be considered in vitro, could bias the response. In any case, the lack of differences in the first stages of fermentation indicates that this was not a major issue, and when expressed as accumulated methane (mmol/g OM) the reduction of particle size increased methane production in the first 24 h of fermentation but only for BP at 12 h and SH at 24 h (significant interaction presentation × substrate, *p* < 0.01).

Despite the range of particle sizes studied, it is worthwhile to consider that Zebeli et al. [14] did not observe differences in VFA concentration in corn silage diets with different proportions of particles below 8 mm; however, they reported a higher fibrolytic enzyme activity as particle size diminished, which agrees with the higher gas production observed in the present work. Yang and Beauchemin [36] did not observe differences in NDF rumen disappearance of corn silage between particles of 5, 11, and 19 mm.

### 4.2. Effect of the Type of Substrate

The substrate that promoted a higher and faster gas production was BP, in response to the high fermentability of its cell wall [30] and the high proportion of soluble fiber, composed by pectins and rapidly fermentable oligosaccharides [37,38]. Afterwards, differences with SH were progressively reduced, whereas that with PK and DA were maintained. This fermentation pattern was supported by total VFA production. The slower fermentation of SH as compared with BP can be explained by its higher NDF proportion, but also by its greater CP fraction. A negative correlation of either feed CP or ammonia released from it and gas production have been reported [39,40], because of both the sequestering of CO_2_ by the released ammonia and the lower fermentable energy of protein as compared with carbohydrates. In this sense, Cone and van Gelder [41] observed that gas production diminishes by 2.5 mL for every percentage unit of CP. Thus, comparing CP of BP and SH should justify around 40 mL lower gas in SH, which is 0.29 to 0.38 of gas differences between these substrates at 12 h incubation. Thereafter, the reduction of differences was possibly due to the exhaustion of both soluble fiber in the case of BP and of protein for SH. The fermentation of both BP and SH promoted a high molar proportion of acetate and propionate [8,42] at the expense of butyrate as compared with the other substrates.

Almost two-thirds of the gas volume from PK and DA was produced during the first 12 h incubation, as in the case of SH mentioned above, the moderately high proportion of CP in both PK and DA in relation to other studied substrates such as BP, BS, and OH could also underestimate their gas production [41], although at a lower extent than for SH. In addition, the high proportion of ether extract in PK can also affect the potential of microbial fermentation, initially in a positive sense because of the rapid use of glycerol from hydrolysis of triglycerides but negatively thereafter by the inhibitory effect of fatty acids on microbial fermentation [43]. The use of glycerol from lipolysis is supported by the high proportion of butyrate and valerate at the expense of acetate, as it has been previously reported [44].

The lowest gas production pattern was observed with both BS and OH, and is explained by their NDF proportion over 760 mg/g. In contrast to other substrates, the highest proportion of gas produced from BS occurred from 12 to 48 h, where 0.72 of total gas volume was released. Similar proportions (0.83 and 0.60, respectively) have been previously recorded [20,45].

In general, the proportion of methane in the total gas produced agreed with the rate and extent of fermentation of substrates. Thus, those that were highly fermented in the first part of incubation (BP, SH, PK, and DA) showed a high proportion of methane in the first 12 h of incubation. The high methane proportion is in contrast with the higher propionate proportion with these substrates, since a negative relationship has been observed among them [46]. In contrast, fermentation of OH, a high fiber, low fermented substrate was less efficient [18] and reached a higher methane proportion in the last part of incubation. In the case of PK, a low methane proportion can be expected because of the effect of its high proportion of ether extract [47]. Because of this inconsistent response among substrates in methane proportion, total methane production per unit of incubated substrate was more dependent on total magnitude of fermentation rather than on qualitative changes in the fermentation pattern. In this regard, no major differences were detected between BS and the other substrates, despite Salami et al. [17] estimated a reduced enteric methane emission when the proportion of non-forage by-products increased. However, despite substrates heterogeneity, a significant correlation was observed between the methane produced per unit of incubated substrate at 12 and 24 h and the NDF content of feeds (*R* = 0.72 and 0.59), although this is of limited value considering the scarce number of substrates tested.

## 5. Conclusions

The particle size of fibrous substrates has a major impact on the extent of their rumen microbial fermentation but not on its rate, and therefore both presentations classify the substrates in the same order. Therefore, their different response in digestive utilization under practical conditions is instead related to factors associated with animal feeding behavior, which is out of the scope of this in vitro work that modulates rumen pH and rate of passage. Methodologically, grinding feeds to 1 mm particle size is a valid standardization in in vitro incubation trials, which does not alter substrates comparison. Fermentation of non-forage fibrous substrates highlights the effects of the proportion and composition of their cell wall fraction. However, other non-fibrous components, such as the levels of protein and fat, can also modulate the response. Methane production as an index of fermentation efficiency did not show major differences between feed presentation forms, nor non-forage substrates as compared with straw and was more related with the magnitude of fermentation than with qualitative changes in fermentation.

## Figures and Tables

**Table 1:** Chemical composition (g/kg DM) and particle size (proportion of particles below a specific size and average size, mm) of feeds used as incubation substrates.

Component	SH	BP	PK	OH	DA	BS
**Chemical composition**						
OM	946	920	962	953	886	872
CP	179	85	157	48	133	61
EE	18	3	88	11	18	13
aNDFom	592	442	555	771	513	760
ADF	417	234	352	376	343	434
ADL	16	23	87	56	66	40
**Particle size**						
<0.15 mm	0.03	0.02	0.05	0.05	0.12	-
0.15–0.30 mm	0.02	0.01	0.06	0.02	0.19	-
0.30–0.60 mm	0.08	0.05	0.25	0.03	0.32	-
0.60–1.20 mm	0.27	0.15	0.18	0.09	0.27	-
1.20–2.00 mm	0.36	0.77	0.46	0.11	0.10	-
>2.00 mm	0.24	0.00	0.00	0.68	0.00	-
average size	1.89	1.77	1.32	5.83	0.83	

SH, soybean hulls; BP, sugarbeet pulp; PK, palm kernel meal; OH, oat hulls; DA, dehydrated alfalfa meal; BS, barley straw; DM, dry matter; OM: organic matter; CP, crude protein; EE, ether extract; aNDFom, neutral detergent fiber; ADF, acid detergent fiber; ADL, acid detergent lignin.

**Table 2:** Methane concentration (mL/mL total gas) at different time intervals, from fermentation of fibrous substrates, non-processed, or in ground form, and organic matter disappearance (OMd, g/g).

Substrate	0–12 h	12–24 h	24–36 h	36–48 h	OMd
Non-processed (NP) SH	0.075	0.187	0.218	0.179 ^ab^	0.827
BP	0.074	0.194	0.187	0.177 ^ab^	0.884
PK	0.076	0.188	0.172	0.162 ^b^	0.573
OH	0.062	0.198	0.207	0.192 ^a^	0.237
DA	0.072	0.198	0.208	0.198 ^a^	0.622
BS	0.060	0.186	0.188	0.182 ^ab^	0.486
Ground (GR) SH	0.081	0.212 ^w^	0.211	0.203 ^w^	0.849
BP	0.078	0.206 ^wx^	0.209	0.187 ^wx^	0.883
PK	0.079	0.180 ^xy^	0.167	0.151 ^y^	0.645
OH	0.060	0.195 ^wxy^	0.203	0.204 ^w^	0.283
DA	0.077	0.209 ^w^	0.202	0.199 ^wx^	0.626
BS	0.052	0.174 ^y^	0.189	0.177 ^xy^	0.481
SEM	0.0040	0.0053	0.0058	0.0060	0.0174
*p*-value Presentation	0.52	0.18	0.90	0.060	0.029
Substrate	<0.001	<0.001	<0.001	<0.001	<0.001
P × S interaction	0.51	0.013	0.15	0.025	0.20

SH, soybean hulls; BP, sugarbeet pulp; PK, palm kernel meal; OH, oat hulls; DA, dehydrated alfalfa meal; BS, barley straw. Means in a column with different superscripts for NP (^a,b^) or GR (^w,x,y^) differ (*p* < 0.05). SEM, standard error of means.

**Table 3:** Average of total volatile fatty acids concentration (VFA, mM) and molar VFA proportions (mmol/mmol) for the main effects’ presentation form and substrate, recorded at 12 h of incubation.

Effect	VFA	Acetate	Propionate	Butyrate	Valerate	BCVFA
Presentation NP	44.8	0.596	0.190	0.164	0.010	0.039
GR	46.5	0.606	0.188	0.159	0.010	0.037
SEM	2.54	0.0132	0.0050	0.0067	0.0006	0.0020
Substrate SH	44.5 ^b^	0.586 ^ab^	0.210 ^ab^	0.147 ^bc^	0.013 ^a^	0.044 ^abc^
BP	68.5 ^a^	0.619 ^ab^	0.238 ^a^	0.123 ^c^	0.007 ^b^	0.014 ^d^
PK	47.4 ^b^	0.531 ^b^	0.169 ^c^	0.254 ^a^	0.011 ^a^	0.035 ^bc^
OH	30.5 ^b^	0.595 ^ab^	0.161 ^c^	0.185 ^b^	0.010 ^ab^	0.050 ^ab^
DA	49.0 ^ab^	0.657 ^a^	0.177 ^bc^	0.126 ^c^	0.009 ^ab^	0.031 ^c^
BS	29.0 ^b^	0.626 ^ab^	0.167 ^c^	0.137 ^bc^	0.012 ^a^	0.059 ^a^
SEM	4.40	0.0164	0.0087	0.0119	0.0010	0.0035
*p*-value						
Presentation	0.40	0.70	0.70	0.49	0.87	0.25
Substrate	<0.001	0.015	<0.001	<0.001	0.002	<0.001
P × S interaction	0.57	0.93	0.86	0.92	0.82	0.54

BCVFA, branched-chain volatile fatty acids (isobutyrate + isovalerate); NP, non-processed; GR, ground; SH, soybean hulls; BP, sugarbeet pulp; PK, palm kernel meal; OH, oat hulls; DA, dehydrated alfalfa meal; BS, barley straw. ^a,b,c^ Means in a column with different superscripts differ (*p* < 0.05). SEM, standard error of means.

**Table 4:** Average of total volatile fatty acids concentration (VFA, mM) and molar VFA proportions (mmol/mmol) for the main effects’ presentation form and substrate, recorded at 24 h of incubation.

Effect	VFA	Acetate	Propionate	Butyrate	Valerate	BCVFA
Presentation NP	61.0	0.606	0.178	0.159	0.014	0.044
GR	68.1	0.613	0.177	0.157	0.014	0.040
SEM	5.24	0.0142	0.0054	0.0066	0.0008	0.0025
Substrate SH	92.9 ^a^	0.663 ^a^	0.173 ^abc^	0.112 ^c^	0.014 ^ab^	0.038 ^ab^
BP	100.7 ^a^	0.632 ^a^	0.208 ^a^	0.125 ^c^	0.010 ^b^	0.025 ^b^
PK	49.4 ^b^	0.505 ^b^	0.157 ^c^	0.278 ^a^	0.017 ^a^	0.042 ^ab^
OH	34.9 ^b^	0.583 ^ab^	0.162 ^bc^	0.184 ^b^	0.015 ^ab^	0.056 ^a^
DA	50.5 ^b^	0.587 ^ab^	0.198 ^ab^	0.149 ^bc^	0.017 ^a^	0.049 ^a^
BS	51.4 ^b^	0.678 ^a^	0.163 ^bc^	0.105 ^c^	0.011 ^b^	0.043 ^ab^
SEM	8.89	0.0240	0.0092	0.0112	0.0014	0.0043
*p*-value						
Presentation	0.42	0.81	0.95	0.83	0.95	0.32
Substrate	0.003	<0.001	0.001	<0.001	0.005	0.001
P × S interaction	0.46	0.54	0.60	0.83	0.19	0.63

^a,b,c^ Means in a column with different superscripts differ (*p* < 0.05). SEM, standard error of means; BCVFA, branched-chain volatile fatty acids (isobutyrate + isovalerate); NP, non-processed; GR, ground; SH, soybean hulls; BP, sugarbeet pulp; PK, palm kernel meal; OH, oat hulls; DA, dehydrated alfalfa meal; BS, barley straw.
